# Dissecting the Role of Bone Marrow Stromal Cells on Bone Metastases

**DOI:** 10.1155/2014/875305

**Published:** 2014-06-26

**Authors:** Denise Buenrostro, Serk In Park, Julie A. Sterling

**Affiliations:** ^1^Department of Veterans Affairs, Tennessee Valley Healthcare System, Nashville, TN 37212, USA; ^2^Center for Bone Biology, Division of Clinical Pharmacology, Department of Medicine, Vanderbilt University, 2215B Garland Avenue, 1235 MRBIV, Nashville, TN 37232, USA; ^3^Department of Cancer Biology, Vanderbilt University, Nashville, TN 37232, USA

## Abstract

Tumor-induced bone disease is a dynamic process that involves interactions with many cell types. Once metastatic cancer cells reach the bone, they are in contact with many different cell types that are present in the cell-rich bone marrow. These cells include the immune cells, myeloid cells, fibroblasts, osteoblasts, osteoclasts, and mesenchymal stem cells. Each of these cell populations can influence the behavior or gene expression of both the tumor cells and the bone microenvironment. Additionally, the tumor itself can alter the behavior of these bone marrow cells which further alters both the microenvironment and the tumor cells. While many groups focus on studying these interactions, much remains unknown. A better understanding of the interactions between the tumor cells and the bone microenvironment will improve our knowledge on how tumors establish in bone and may lead to improvements in diagnosing and treating bone metastases. This review details our current knowledge on the interactions between tumor cells that reside in bone and their microenvironment.

## 1. Introduction

**Figure 1:**
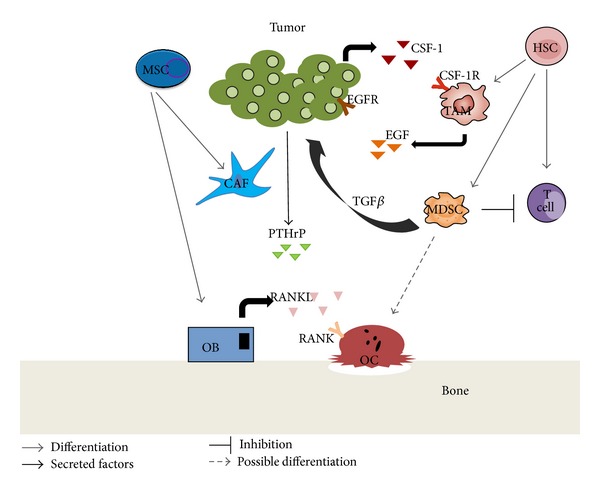
Tumor microenvironment interactions. Tumor cells interact with the cell populations present in the bone marrow. These include cells such as the fibroblasts, osteoblasts, osteoclasts, immune cells, and others as depicted here.

Despite recent advances in early detection and therapeutic approaches, metastases still remain the major problem for cancer patients. In particular, bone metastases account for decreased quality of life and ultimately death of prostate, breast, and lung cancer patients. However, current therapeutic approaches are insufficient to effectively cure or prevent bone metastasis. Tumor metastasis is a tightly regulated multistep process, in which specific interactions between disseminating tumor cells and the cells constituting the recipient organ microenvironment play important roles. Increasing evidence supports the prometastatic functions of the microenvironment, with many studies indicating the importance of bone marrow cells in the metastatic niche. Many early studies have shown that these bone marrow cells set up a metastatic niche at the secondary site that allows for cells to establish [1, 2]. Subsequent studies have specifically isolated myeloid-derived suppressor cells [3–6], myofibroblast [7–9], and tumor-associated macrophages [10–12]. Each of these has some overlapping roles in metastasis, but each class of cells is a distinct bone marrow cell type with distinct roles in metastasis (summarized in Figure 1). While these classes of cells were isolated and shown to be important in metastases, many groups are still actively trying to clarify their precise molecular role in the metastatic process. Researchers expect that advanced knowledge on how these cells regulate the tumor microenvironment will allow development of novel therapeutic approaches to alter the niche less hospitable to the cells and therefore reducing or preventing tumor growth. It is also possible that understanding the niche will allow clinicians to better predict which patients may develop secondary disease and which organs may be affected.

## 2. Bone Cells

The importance of interactions between tumor cells and other cells in the bone microenvironment was demonstrated in the 1990s by the work of Dr. Greg Mundy and others in the field. Their work strongly showed that there was a vicious cycle between the tumor cells and cells in the bone microenvironment. This work showed that tumor cells secreted factors that stimulated bone destruction, while bone destruction caused the release of growth factors from the bone matrix that further stimulated the tumor cell growth and production of factors that further enhanced bone destruction [13–15].

### 2.1. Osteoclasts

Osteoclasts are multinucleated cells that are responsible for bone resorption. A functional osteoclast has the ability to resorb mineralized bone matrix as part of normal bone remodeling that occurs during an individual's lifetime [16, 17]. Osteoclasts differentiate from myeloid progenitor cells under the influence of growth factors and cytokines such as macrophage colony stimulating factor (M-CSF) and receptor activator of nuclear factor kappa-B ligand (RANKL) [18, 19]. Physiological bone resorption is a tightly regulated process that involves signals from osteoblasts as well as signals from other cells found in the microenvironment. Osteoclast differentiation, maturation, and activation are dependent on RANK/RANKL/osteoprotegerin (OPG) signaling pathway [20, 21]. OPG is a soluble decoy receptor for RANKL, expressed by osteoblasts and negatively regulates osteoclast activation [19, 21, 22]. Deregulation of this process, such as too much resorption or too little, can lead to increased risk of fracture as well as other bone-related diseases [17].

Overactive osteoclasts can be detrimental and play a role in several diseases such as osteoporosis, pycnodysostosis, and Paget's disease, which occur due to increased bone resorption and bone loss [23]. Primary cancers of breast, lung, and prostate cancer have a propensity to metastasis to bone [22, 24–26]. These cancers cells secrete factors such as parathyroid hormone-related protein (PTHrP) which stimulate osteoclast-mediated bone destruction through the RANK/RANKL/OPG signaling pathway [22, 27]. During bone destruction growth factors including transforming growth factor beta (TGF-*β*), insulin-like growth factors (IGFs), and others are released from the bone matrix, which can stimulate further tumor growth and the production of tumor-derived factors (such as PTHrP) that can stimulate further bone destruction [15, 22, 28].

Even though much is known about the role of osteoclasts within the vicious cycle, many of their functions are yet to be explored. Increased osteoclast activity can be due to several different factors but the end results seem to be the same, which is that over activation of these cells promotes osteolysis and tumor cell growth, because factors released from bone during resorption stimulate tumor cell proliferation. CXCR4 is found on osteoclast precursors and regulates hematopoietic and tumor cell homing to bone. In studies where mice were reconstituted with* Cxcr4* ^−/−^ hematopoietic cells had increased bone resorption and bone loss, specifically* Cxcr4* ^−/−^ osteoclasts had higher resorptive activity and faster differentiation compared to control osteoclasts. The authors concluded that because the reconstituted mice had increased tumor growth in bone compared to control mice that disruption in CXCR4 may increase osteoclastogenesis leading to increased resorption and tumor burden [29]. A recent paper published by Ell et al. showed that mice injected with pre-miR-141 and pre-miR-219 had reduced osteoclastic activity and osteolytic bone metastasis [30].* Src* ^−/−^ mice have shown impaired osteoclast functions [31, 32], and Src inhibitors have shown to suppress bone resorption effects [33]. These data suggest that Src may be an ideal therapeutic target to suppress tumor cells (frequently expressing high Src activities) and osteoclasts (requiring Src for function) at the same time [34, 35]. However, recent phase III clinical trial results showed that addition of dasatinib (a Src family kinase inhibitor) to docetaxel did not significantly improve overall survival of castration-resistant prostate cancer patients [36]. Araujo et al., the lead investigator of the failed clinical trial, pointed out that further understanding of Src inhibitors' mode of action could identify a better therapeutic role, because the clinical trial included heterogeneous patient population [36].

Inhibition of osteoclast activity by a variety of different factors has shown to decrease tumor burden in a mouse breast cancer bone metastasis model [37]. The most commonly used class of osteoclast inhibitors includes bisphosphonates, which bind to the bone promoting osteoclast apoptosis and inhibiting osteoclast mediated bone resorption [38]. Bisphosphonates (including zoledronate, alendronate, ibandronate, etc.) have been highly successful for reducing skeletal related events in patients with osteoporosis and with tumor-induced bone destruction [39, 40].

Alternatively, RANKL inhibitory antibodies have been promising both clinically and in preclinical models where they can increase time to skeletal related events (SRE) [41, 42]. Denosumab (Prolia, XGEVA), a monoclonal antibody against RANKL, was recently demonstrated to significantly increase time to SRE compared to a bisphosphonate, zoledronic acid, in breast and prostate cancer patients with bone disease [43, 44]. The debate between clinicians regarding which treatment is more efficacious continues, but both options are clearly effective and have their benefits. One concern regarding both treatments is the serious, yet rare, side-effects such as atypical fractures and osteonecrosis of the jaw [25, 37, 38]. Additionally, neither treatment has been shown to cure bone metastases or significantly increase survival in patients with bone metastases.

### 2.2. Osteoblasts

Osteoblasts are mesenchymal-origin cells lining the endosteal surface of bone and constitute approximately 4–6% of all bone cells. Osteoblasts produce organic matrix of bone and subsequently deposit inorganic components (e.g., calcium and phosphate), resulting in mineralized hard tissue. In addition to their physiologic functions, osteoblasts are important components of the metastatic bone microenvironment. The best-characterized role of osteoblasts in bone metastasis is described in the “vicious cycle hypothesis” where osteoblasts produce M-CSF and RANKL, two essential factors for osteoclastogenesis [14, 27, 45]. Subsequent studies followed to understand how molecular alterations in osteoblasts contribute to create a congenial microenvironment for metastatic tumor cells. Schneider et al. demonstrated that expansion of osteoblasts by administration of bone-anabolic agents such as parathyroid hormone (PTH) increased prostate tumor cell localization and growth in bone [46], suggesting that higher bone turnover rates (i.e., increased activity and number of osteoblasts) are associated with bone metastasis. Other studies have suggested that osteoblasts can function as a prometastatic population of cells. The first experimental evidence to support this come from the physiological phenomena of hematopoietic stem cell (HSC) homing in bone. HSCs migrate and repopulate the bone marrow immediately after birth, while the liver is the primary site of hematopoiesis during feral development. Taichman et al. demonstrated that CXCL12/SDF-1 (expressed by osteoblasts and endothelial cells) and its receptor (CXCR4, expressed by prostate cancer cells) regulate bone-tropism of prostate cancer cells [47]. In addition to the CXCL12/CXCR4/CXCR7 axis [48], Annexin II, expressed by osteoblasts and endothelium, regulates HSC adhesion, homing, and engraftment [49]. More recently, Jung et al. demonstrated that differential levels of growth arrest specific- (GAS-) 6 protein in the bone stromal cells (dominantly osteoblasts) induce metastatic tumor cell dormancy and determine site-specificity (i.e., increased localization in vertebrae and hind limb long bone compared with fore limb bones) of murine experimental metastasis model of human prostate cancer [50]. Furthermore, the same group provided pivotal evidence that osteoblastic niche for HSC is the direct target of tumor cell localization in bone [51]. The authors demonstrated that increasing the HSC niche size (via administration of PTH to induce osteoblast proliferation) promoted skeletal localization of prostate cancer cells, while decreasing the niche size (via conditional ablation of osteoblasts) reduced tumor cell localization. The author further investigated whether HSC compete with metastatic cancer cells for occupancy in the bone marrow. Administration of AMD3100 (a clinical regimen to mobilize HSC) mobilized metastatic cancer cells in the niche back into the circulation, indicating that HSC compete with bone-tropic cancer cells. These data collectively suggest that adhesion molecules and chemokine/chemokine receptors expressed on osteoblasts contribute to localization and subsequent growth of metastatic tumor cells in bone.

Increasing evidence supports that osteoblastic cells contribute to the metastatic progression by releasing cytokines and growth factors in the microenvironment. We have recently demonstrated that primary prostate tumor cells distantly instigate osteoblasts (via PTHrP in the systemic circulation) to increase vascular endothelial growth factor- (VEGF-) A, interleukin- (IL-) 6, and C-C chemokine ligand- (CCL-) 2 in the bone microenvironment and that VEGF-A and IL-6 in turn stimulate myeloid-derived suppressor cells with increased angiogenic potentials [52]. Indeed, hematopoietic lineage cells are dependent on bone cells (predominantly osteoblastic cells) for proliferation, mobilization, and function. This concept of “osteoimmunology” is now expanding to the role of osteoblasts in regulating other adjacent bone marrow cells (e.g., hematopoietic lineage cells with prometastatic functions, such as myeloid-derived suppressor cells). Interestingly, those prometastatic cytokines (in particular, VEGF-A and IL-6) stimulate osteoblasts to produce more VEGF-A and IL-6, suggesting that osteoblastic cells may function as an amplification mechanism of cytokines in the bone microenvironment.

## 3. Immune Cells

### 3.1. Myeloid Derive Suppressor Cells

The role and existence of myeloid derived suppressor cells (MDSCs) have been quite controversial among scientists since their initial discovery in 1978 [53]. Initially they were recognized as natural suppressor cells located in the bone marrow and spleen that were able to suppress cell-mediated immunity [54]. These cells did not contain cell surface markers that resembled T cells, B cells, macrophages, or natural killer cells which made it difficult to phenotypically characterize them [55, 56]. MDSCs are a heterogeneous population of myeloid cells that are at different stages of differentiation. This population includes immature macrophages, granulocytes, and dendritic cells as well as myeloid progenitor cells [5, 57, 58]. In mice these cells can be characterized into two major subtypes, monocytic-MDSCs and granulocytic-MDSCs, through lymphocyte antigens Ly6C and Ly6G [59]. Both subtypes have immune suppressive functions that are regulated through distinct mechanisms. Granulocytic-MDSCs have been found to express higher levels of ROS (reactive oxygen species) and low levels of NO (nitric oxide) verses monocytic-MDSCs expressing higher NO and lower ROS expression [59, 60]. Suppressive MDSCs are not found in healthy hosts; only their nonsuppressive counterpart iMCs (immature myeloid cells) are present. MDSCs need to be activated to express suppressive function and are only present at sites of chronic pathological conditions such as infection and cancer [53].

Recently these cells have been recognized to play an important role in tumor progression in many solid tumors by inhibiting antitumor immune responses and by promoting tolerance [58, 61]. These cells have been deemed protumorigenic due to their suppression of T cells, promotion of angiogenesis, invasion, and metastasis [5, 6, 53, 62]. MDSCs have been directly linked to promoting tumor invasion and metastasis through the production and secretion of factors such as MMPs, IFN*γ*, IL-10, and TGF-*β* [6, 61]. They have also been known to suppress the immune system by promoting tolerance by accumulating T regulatory cells [58, 61, 63]. In cancer, MDSCs are activated by tumor-secreted factors such as Toll-like receptors (TLRs), IL-4, IL-13, and TGF-*β* that activate several different signaling pathways [64]. Specific MDSC expansion in the tumor microenvironment is guided through tumor-derived factors and factors from the microenvironment that is context specific dictating which population (monocytic versus granulocytic) is increased [46].

The presence and accumulation of MDSCs has been well reported in several human cancers as well as different disease types in the last several years. A positive correlation between stage and MDSC peripheral density has been reported in both melanoma and head and neck squamous cell carcinoma (HNSCC) patients [65]. A 15 percent increase in circulating CD14^+^ HLA-DR^−/lo^ cells was correlated with advanced stage (III and IV) as compared to early stage (I and II) HNSCC patients [65]. MDSCs containing the phenotype LIN^−^HLA-DR^−^CD33^+^CD11b^+^ have been isolated from the blood of patients with glioblastoma, breast, colon, lung, and kidney cancers [58, 62, 66, 67]. MDSCs containing the phenotype CD11b^+^CD14^−^HLA-DR^−/low^CD33^+^CD15^+^ were found in the bone marrow and the peripheral blood of patients with active multiple myeloma compared with healthy donors [68].

The role that MDSCs play in human tumor-induced bone disease is still relatively unknown. With the use of mouse models, several published papers have demonstrated that MDSCs play an important role in bone metastasis. This is consistent with what is known about MDSC's contribution in the primary tumor environment. What is unknown is if MDSCs perform a direct role in promoting tumor establishment or tumor proliferation in bone by assisting the tumor itself or indirectly by secreting protumorigenic factors that prime the bone allowing it to become a hospitable host. Published papers have used mouse models to show that MDSCs can promote tumor growth in bone [52, 57, 69]. In a prostate cancer mouse model, it was demonstrated that tumor-derived PTHrP indirectly increases MDSC's angiogenic potential therefore contributing to tumor growth and angiogenesis [52]. Danilin and colleagues showed that MDSCs contribute to breast cancer osteolysis by inducing expression of Gli2 and PTHrP in tumor-bearing mice. These factors stimulate osteoclast-mediated bone destruction leading to increased bone lesions compared to control mice [57]. This group also showed that MDSCs isolated from tumor-bearing mice had the potential to differentiate into osteoclasts* in vitro* and* in vivo* [57]. Sawant et al. published this as well and explained that the reason MDSCs could differentiate into osteoclasts is because they are novel osteoclast progenitors driving bone metastasis during cancer progression [69].

MDSCs as a potential therapeutic target have been the topic of discussion since their identification. Studies have shown that eliminating MDSCs increases immune-surveillance and decreases tumor growth [63, 70, 71]. There are many different ways to target MDSCs including growth factors (anti-VEGF antibodies), chemokines (anti-CCL2 antibodies), cytotoxic drugs (Gemcitabine), enzyme inhibitors (amino-bisphosphonate), signaling inhibitors (sunitinib), and inducing differentiation (ATRA-All-trans retinoic acid) [72]. Src inhibitors have shown promise in targeting MDSCs by inhibiting their recruitment and MMP-9 gene expression in the tumor microenvironment [73]. Gemcitabine is a nucleoside metabolic inhibitor used to treat several types of cancers and has been shown to decrease MDSC levels in tumor-bearing mice by inhibiting expansion [74, 75]; however, its precise mechanism of MDSC inhibition is not fully understood. Bisphosphonates, which are routinely prescribed for cancer patients with bone metastasis, have also been demonstrated to decrease MDSC expansion in tumor-bearing mice through the reduction of MMP-9 expression [76]. Additionally, STAT3 inhibitors have also been successful at targeting MDSC in preclinical models [65]. While more studies are needed to understand the mechanisms of action, it is clear that targeting MDSCs clinically is both possible and promising therapeutically.

### 3.2. Tumor-Associated Macrophages

Macrophages are professional phagocytes that are differentiated from the myeloid lineage and are identified by the expression of certain markers as well as by the phenotypic differences among them [77, 78]. They have roles in development, homeostasis, tissue repair, and immunity and have been linked to many diseases including cancer [77, 78]. These are plastic cells and their phenotype is consistently modulated by the local microenvironment [79]. Macrophages can be classified by their immunological responses such as classically activated macrophages (M1) that are involved in inflammatory responses and alternatively activated macrophages (M2) that are involved in wound healing [77, 78, 80, 81]. M2 macrophages have been implicated in having protumor properties due to the cytokines, chemokines, and growth factors that they release such as VEGF, IL-10, TGF-*β*, EGF, and MMPs, among many others [77, 82]. These protumor macrophages are referred to as tumor-associated macrophages (TAMs) and are considered to be phenotypically similar to M2 macrophages [81, 83, 84].

Macrophage growth, chemotaxis, and differentiation are controlled by several chemokines including CCL-2 (also known as monocyte chemoattractant protein [MCP]-1) and growth factors such as CSF-1 [77]. CSF-1 is the regulator of the differentiation, proliferation, and survival of macrophages and their precursors [85]. CSF-1 overexpression has been implicated in the poor prognosis of several cancers and is currently being investigated as a possible therapeutic target [85–89]. In an invasive breast cancer mouse model, macrophages have been implicated in assisting tumor cell motility by participating in an epidermal growth factor- (EGF-) CSF-1 paracrine loop where tumor cells secrete CSF-1 and macrophages contain the corresponding receptor and vice versa [66, 67, 69, 75]. CCL2 is a chemokine that has been implicated in assisting cancer metastasis by mediating a crosstalk between cancer cells and the stromal cells that are present in the tumor microenvironment [90]. CCL2 is expressed by many tumor types as well as by the peripheral myeloid population [91]. Roca and colleagues showed that CCL2 stimulation induces peripheral blood monocytes to differentiate to M2 macrophages compared to unstimulated control monocytes [91].

In several papers macrophages have been reported to promote tumor initiation, progression, invasion, and metastasis [77, 79, 84]. Activated macrophages produce inflammatory factors such as reactive oxygen and nitrogen species in response to signals from other immune cells creating a constant inflamed stromal environment [80]. Chronic inflammation generates a stromal environment susceptible to mutations and has been linked to tumor initiation and growth. Progression of a mass from a neoplasia/adenoma to an early carcinoma is prompted through their secretion of VEGF and other angiogenic factors stimulating angiogenic switch [80]. Several groups have shown that an increase in macrophage density correlates with poor patient prognosis and survival in thyroid, lung, breast, and hepatocellular cancers [84, 89, 92, 93]. However, in other cancers such as stomach, colorectal, and pancreatic cancer, a high macrophage density is correlated with a good patient prognosis [80, 94].

The role of macrophages at the primary site is well established but their function at distant metastatic sites is still being highly investigated. Myeloid derived cells have been found to accumulate at distant sites priming the environment for tumor colonization [1, 2]. This notion of a premetastatic niche has been around for several years and has been found to be important in the primary site but has yet to be proven to exist in bone. This theory encompasses that once there is an established primary tumor site, hematopoietic progenitor cells are signaled to migrate from the bone marrow into secondary metastatic sites, such as the lung, and alter the microenvironment leading to activation of integrins and chemokines that promote attachment, survival, and growth of tumor cells [1]. Proving that this process occurs in bone has been challenging because hematopoietic progenitor cells originate in the bone marrow and do not have to migrate to reach the bone microenvironment. It is more likely that in bone microenvironment stromal cells including macrophages are “reeducated” by tumor-derived factors and begin priming the bone before tumor establishment occurs.

Several therapeutic approaches to target macrophages have been explored. One approach includes the inhibition of TGF-*β* signaling, which was demonstrated through preclinical studies by deleting TGF*β* type 2 receptor (RII) in the macrophages. These studies demonstrated that animals with RII deficient macrophages displayed a reduction in tumor growth due to decreased secretion of myeloid factors that assist in tumor progression [82, 95]. Other therapeutic approaches target macrophage factors such as CSF-1 and its receptor [85, 87]. Currently, in clinical trials are small molecules and monoclonal antibodies that inhibit CSF-1 and prevent its binding, or the tyrosine kinase activity [96]. Other therapeutic strategies include preventing the recruitment of macrophages through inhibition of inflammatory monocyte trafficking with anti-CCL2 or CCR2 antibodies [96]. However, a recent phase II clinical trial for carlumab (anti-CCL2 monoclonal antibody) in metastatic prostate cancer patients did not support antitumoral activity as a single agent (PMID 22907596). Since TAMs, macrophages that have been educated by the tumor cells and assist in cancer progression have been implicated in causing resistance to tamoxifen in breast cancer and to androgen receptor antagonists in prostate cancer; a potential future therapeutic strategy could be to reeducate TAMs to express an antitumor phenotype that would work against the tumor instead of with it [79, 80, 83, 84].

### 3.3. Other Immune Cells

The bone marrow is a rich environment for many different immune cells including the B-cells, T-cells, and NK-cells, all of which are known to be important in cancer progression and soft tissue metastases [97]. Yet despite their proximity and abundance in bone metastases, relatively few studies have been performed to investigate their role in tumor-induced bone disease. This is in part due to the fact that the vast majority of bone metastasis studies utilize human tumors in immune-deficient mice, most commonly these models of T-cell deficient mice, but other models are also lacking B-cells (SCID,* Rag 2*−/−,* Rag 1*−/−). This makes understanding the role of T- and B-cells in bone metastases challenging.

T-cells are well-known to inhibit tumor growth, and in line with this finding it has been shown that stimulating T-cell response in mice reduced tumor burden in bone while reducing it blocks tumor growth in bone [98]. However, a recent study demonstrated that tumor associated T-cells can induce osteolytic bone disease prior to bone colonization. In this study they show that T-cell produced RANKL can induce osteoclastogenesis and bone destruction [99]. These data suggest that T-cells may have a dual role in bone disease in that they can reduce tumor growth but stimulate bone destruction. Regardless, since the majority of cancer and bone studies utilize T-cell deficient mice, it is clear that tumor cells can grow and metastasize to bone in the absence of T-cells.

Much less information exists describing the interactions between tumors in bone and B-cells or NK cells. A few manuscripts describe interactions between NK cells and tumors in bone. Specifically, they show that inhibiting NK cells increases tumor take in animal models of prostate cancer [100]. Other papers describe that NK cells are reduced in prostate cancer [101] but that forced expression of NK associated ligands can reduce tumor growth [102, 103]. Another immune lineage cell that has been implicated in cancer induced bone disease is the Megakaryocytes. Li et al. demonstrated that megakaryocytes could reduce prostate tumor cell growth and increase apoptosis, while their expansion* in vivo* reduced tumor-induced bone destruction [104].

## 4. Cancer-Associated Fibroblast (CAF)

Fibroblasts are another cell type that is abundant in the bone marrow microenvironment. CAFs are defined as fibroblast that reside in the tumor mass and are capable of promoting tumor growth. These cells are typically myofibroblast-like cells that express *α*-Smooth muscle actin (*α*-SMA), vimentin, and fibroblast specific protein-1 (FSP1) [105]. Some of the early studies showed that these fibroblasts could be recruited from the bone marrow to the tumor [106] and that they could stimulate malignant transformation [7], tumor cell growth, and invasion [107]. These effects on tumor cell growth are thought to be mediated through CXCL12 [108] and TGF-*β* [109]. Other pathways including Wnt signaling [110], bone morphogenetic proteins [111], and MMPs [112] have also been associated with their invasive potential. Other papers have demonstrated that in addition to factors secreted by fibroblasts that they can induce a more invasive phenotype through physical properties as well. One study showed that the increase in fibroblasts increased the stiffness of the tumor, which can activate pathways within the tumor cells that induces a more invasive phenotype [113]. Interestingly, our previous publications have demonstrated that rigidity influences gene expression in tumor cells [114]. Taken together, this suggests that rigidity may also influence expression in the fibroblast and further contribute to tumor-induced bone disease.

In addition to regulating invasiveness of the primary site, other studies have investigated the role of CAFs in the establishment of secondary sites. For example CAFs have been shown to be recruited to sites of liver metastases in colon cancer [115]. Additionally, CAFs have been associated with bone metastases, in which the loss of TGF-*β* receptor type II (RII) in the CAFs stimulated prostate cancer cell growth in the bone. More importantly, a recent paper by Joan Massague's group demonstrated that CAF content in triple negative tumors was associated with bone metastases, but not lung, in patient samples [9].

Because of their association with tumor growth, invasion, and metastasis, CAFs make a compelling target for the development of therapeutics. This is also compounded by the fact that CAFs have been associated with chemotherapeutic resistance [116–118]. One group found that CAF-induced resistance to tamoxifen could be reversed using metformin or arsenic trioxide [119]. Other groups tried to target fibroblast activating protein using an anti-FAP antibody (sibrotuzumab) in clinical trials of metastatic colorectal cancer, but these studies showed no significant efficacy [120–122]. However, the use of FAP conjugated therapies has been shown to increase drug efficacy and reduce side-effects associated with chemotherapy [123, 124].

## 5. Mesenchymal Stem Cell (MSC)

MSCs are a pluripotent population of bone marrow cells that can differentiate with many different cell types, including osteoblasts, adipocytes, chondrocytes, and fibroblasts. Similar to CAFs, MSCs have been shown to associate to sites of tumor in many different tumor types [125] and have been demonstrated to promote proliferation and migration [9, 126, 127]. In Massague's recent paper, they showed that MSCs induced a transcriptional shift in tumors similar to CAFs and that MSCs could recapitulate the CAF phenotype [9]. This suggests that in breast cancer CAFs and MSCs function similarly. However, unlike CAFs, MSC association with tumors has not been completely associated with negative outcomes, and in some cases MSCs may inhibit tumor growth. In fact, in some malignancies, such as multiple myeloma (MM), MSCs are used therapeutically. Some treatments for MM involve cell-based therapies in which patients are given autologous stem cell transplants, under the reasoning that this may recapitulate normal immune cells that may fight the disease [128]. A recent myeloma study suggests that using MSCs with high Fas ligand in multiple myeloma bearing mice increased apoptosis of the myeloma cells [129]. Since MSCs “track” to tumors some groups have developed modified MSCs as cargo for the delivery of therapeutics [125], but due to treatment concerns they have not been tested clinically. Clearly more needs to be understood about MSCs and how to select for more specific populations.

## 6. Conclusions

The bone microenvironment is a rich milieu of different cell types, with each having a specific role on tumor cells both that metastasize to the bone and to other sites. While the past decade has seen an increase in research devoted to understanding the role of each cell-type in different malignancies, there are still many questions. In reality it is likely that these cells work together to regulate tumor growth, invasion, and metastasis and that new approaches need to be undertaken to study the complex interaction that occur between these multiple cell types. Many groups are beginning to collaborate with systems biologists, engineers, and computer scientists to investigate these interactions in a more comprehensive manner. Once we better understand these interactions, more possibilities of therapeutic interventions will become possible.
